# Modulation of NF-κB/miR-21/PTEN Pathway Sensitizes Non-Small Cell Lung Cancer to Cisplatin

**DOI:** 10.1371/journal.pone.0121547

**Published:** 2015-03-23

**Authors:** Zhenhua Yang, Surong Fang, Yicheng Di, Weiwei Ying, Yan Tan, Wei Gu

**Affiliations:** Department of Respiratory Medicine, Nanjing First Hospital, Nanjing Medical University, Nanjing, China; IPMC, CNRS UMR 7275 UNS, FRANCE

## Abstract

**Background:**

Platinum-based chemotherapy is a standard strategy for non-small cell lung cancer (NSCLC), while chemoresistance remains a major therapeutic challenge in current clinical practice. Our present study was aimed to determine whether inhibition of the NF-κB/miR-21/PTEN pathway could increase the sensitivity of NSCLC to cisplatin.

**Methods:**

The expression of miR-21 in NSCLC tissues was determined using in situ hybridization. Next, the effect of miR-21 on the sensitivity of A549 cells to cisplatin was determined in vitro. Whether miR-21 regulated PTEN expression was assessed by luciferase assay. Furthermore, whether NF-κB targeted its binding elements in the miR-21 gene promoter was determined by luciferase and ChIP assay. Finally, we measured the cell viability and apoptosis under cisplatin treatment when NF-κB was inhibited.

**Results:**

An elevated level of miR-21 was observed in NSCLC lung tissues and was related to a short survival time. Exogenous miR-21 promoted cell survival when exposed to cisplatin, while miR-21 inhibition could reverse this process. The RNA and protein levels of PTEN were significantly decreased by exogenous miR-21, and the 3′-untranslated region of PTEN was shown to be a target of miR-21. The expression of miR-21 was regulated by NF-κB binding to its element in the promoter, a finding that was verified by luciferase and ChIP assay. Hence, inhibition of NF-κB by RNA silencing protects cells against cisplatin via decreasing miR-21 expression.

**Conclusion:**

Modulation of the NF-κB/miR-21/PTEN pathway in NSCLC showed that inhibition of this pathway may increase cisplatin sensitivity.

## Introduction

Non-small cell lung cancer (NSCLC), comprising squamous cell carcinoma, adenocarcinoma and large cell undifferentiated carcinoma, is the most common type of lung cancer [1,2]. Genetics plays an essential role in the pathophysiological mechanism of NSCLC [3,4], and it leads to non-sensitivity of NSCLC to platinum-based chemotherapy [5], resulting in NSCLC being the most common cause of cancer-related mortality worldwide [1]. Thus, it is necessary to develop novel drug targets based on molecular genetics.

MicroRNAs (miRNA) have been implicated as key modulators of multiple target genes through the endogenous RNA interference machinery [6]. They contribute to cancer biology by altering the expression of their target genes [7]. miR-21 is one type of miRNA that functions as a potent modulator of tumor cell behavior and malignant transformation [8]. It has been found to increase cell growth in liver cancer and has demonstrated anti-apoptotic properties in glioblastoma [9]. In NSCLC, miR-21 also plays a fundamental role in cellular proliferation, invasion and apoptosis [10]. Previous studies have demonstrated that miR-21 regulates the expression of phosphatase and tensin homolog (*PTEN*) via directly targeting its 3′ untranslated region (3′UTR) [8]. *PTEN*, a tumor suppressor gene, plays an essential role in the regulation of the cell cycle, apoptosis and formation of many types of solid tumors, including NSCLC [10]. It negatively regulates the intracellular levels of phosphatidylinositol-3,4,5-trisphosphate (PI3K) in cells and functions as a tumor suppressor by negatively regulating the Akt signaling pathway [11]. Under normal conditions, the expression and activation of *PTEN* is tightly controlled, whereas the expressions of miRNAs are dysregulated in NSCLC. Alteration of *miR-21/PTEN* was found in NSCLC patients with gefitinib resistance [12].

Given the essential role of the miR-21/PTEN pathway in NSCLC, there remains a question regarding why the expression of *miR-21* is increased in NSCLC. We performed a bioinformatics search (http://alggen.lsi.upc.es/cgi-bin/promo_v3/promo/promo.cgi) and found four binding elements of NF-κB in the promoter of the *miR-21* gene. Additionally, one research group demonstrated that DNA damage induced *miR-21* upregulation and promoted breast cancer cell invasion in an NF-κB-dependent manner [13]. Hence, we assumed that NF-κB increased the level of *miR-21*, which reduced *PTEN* expression post-transcriptionally to promote cell survival under cisplatin treatment in NSCLC; therefore, inhibition of the NF-κB/miR-21/PTEN pathway might be a potential drug target for NSCLC.

## Methods

### Tissue microarray

Tissue microarray (TMA) was prepared from tissues derived from 34 patients with NSCLC and 10 patients without NSCLC between January 1999 and August 2007. The study was conducted in accordance with the Declaration of Helsinki (1989) and was approved by the local Ethics Committee (Nanjing First Hospital, Nanjing Medical University, Nanjing, China). Written informed consent was obtained from each patient. The representative area was carefully selected from a hematoxylin and eosin (HE)-stained section, and then a 1.5 mm tissue core was obtained from the corresponding paraffin blocks. Thereafter, paraffin-embedded material was cut into 5 μm sections and placed onto a slide, followed by staining with HE or in situ hybridization (ISH). Sections were viewed under a light microscope (Olympus, Japan). NSCLC was diagnosed by two independent pathologists according to the Union for International Cancer Control (UICC) classification system, 7th edition. The staining intensity was scored as follows: 0, no staining of cells; 1, weak staining; and 2, strong staining. The percentage of stained cells was classified using a 3-grade scale: 0, no positive cells; 1, <50% positive cells; and 2, >50% positive cells.

### ISH

ISH of *miR-21* was performed according to the manufacturer’s protocol (MK10013, Boster, China). Briefly, after deparaffinization of slides in xylene and ethanol, slides were incubated with 3% H_2_O_2_ for 10 min at room temperature and then digested with pepsin for 10 min at 37°C. After rinsing in water, slides were fixed with 1% PFA in DEPC (Generay, China) for 10 min. Sections were then washed in water three times and incubated with prehybridization buffer for 4 h at 42°C and hybridization buffer overnight at 42°C with an miR-21 probe (5’-TAGCTTATCAGACTGATGTTGA-3’). Slides were washed as follows: 2×SSC for 5 min at 37°C, twice; 0.5×SSC for 15 min at 37°C, once; and 0.2×SSC for 15 min at 37°C, once. The tissue was incubated in blocking buffer at 37°C for 30 min and then was incubated with streptavidin-biotin complex (SABC). After washing three times with PBS, slides were incubated with horseradish peroxidase polymer conjugate for an additional 30 min at room temperature. Subsequently, they were stained with 3,3-diaminobenzidine and counterstained with hematoxylin (Sigma-Aldrich, US).

### Cell culture

HEK293 cells and A549 cells were grown and maintained in Dulbecco’s modified Eagle’s medium (DMEM, Invitrogen, US) supplemented with 10% fetal bovine serum (FBS, Gibco, US), penicillin 100 U/ml and streptomycin 100 μg/ml (Gibco).

### Construction of plasmid and recombinant lentivirus

The human *NF-κB* (p65) gene fragment amplified by PCR was cloned into pcDNA3 and the insertion was confirmed by sequencing. Then, the plasmid pcDNA-NF-κB was digested by EcoRI/XbaI. The digested products were electrophoresed in an agarose gel. The *NF-κB* gene fragment was purified by gel extraction and subsequently cloned into pLVX-IRWS-ZsGreen1. An shRNA construct targeting NF-κB was also cloned into pLVX-IRWS-ZsGreen1. Recombinant lentiviruses Lenti-shNF-κB and Lenti-NF-κB were produced and titrated according to a previous report [14]. The entire open reading frame of *PTEN* with or without the *miR-21* targeting sequence in the 3′UTR was cloned into the pcCS2 vector with a Myc tag to construct pcCS2-PTEN-3’UTR and pcCS2-PTEN, respectively.

### Transfection of *miR-21* mimics and inhibitor

The FAM-modified 2′-O-Me-oligonucleotides were chemically synthesized and purified by high-performance liquid chromatography (GenePharma, China). The 2′-O-Me-miR-21 mimic was composed of RNA duplexes with the following sequence: 5′-UAGCUUAUCAGACUGAUGUUGA-3′; the sequences of 2′-O-me-miR-21 inhibitor and 2′-O-me-scrambled oligonucleotides were as follows: 5′-UCAACAUCAGUCUGAUAAGCUA-3′; and 5′-CAGUACUUUUGUGUAGUACAA-3′. The cells were seeded on a 24-well plate at a density of 5×10^4^ cells per well and grown to 80% confluence. The miR-21 inhibitor or miR-21 mimics at a concentration of 50 nM each was transfected into cells using Lipofectamine 2000 (Invitrogen, USA) by incubation in Opti-Mem I media (Invitrogen) for 4 h. The cells were then transferred into fresh culture medium. After incubation for 24 h, the culture medium was replaced and fluorescent images were acquired to monitor the transfection efficiency. After 48 h, cells were harvested for analysis.

### Real-time PCR

Total RNA was isolated from cells using Trizol reagent (Invitrogen) following the manufacturer’s recommendation. For *miR-21* qRT-PCR, total RNA was reverse transcribed using an miRNA-specific primer and the miScript Reverse Transcription kit (Qiagen, Germany). Stem-loop qRT-PCR was performed using the SYBR green PCR Master Mix (Qiagen) according to the manufacturer’s protocol. Relative expression was evaluated by the comparative Ct method and normalized to the expression of U6 small nuclear RNA. For the detection of *PTEN*, reverse transcription was carried out using the First-strand cDNA synthesis kit (Promega, US) following the manufacturer’s recommendations. qRT-PCR was performed using the QuantiFast SYBR Green PCR Kit (Qiagen). The *GAPDH* mRNA level was employed as an internal control. All of the samples were analyzed in triplicate in three independent experiments. Reactions without cDNA were used as the no-template control, and no-RT controls were also set up to exclude genomic DNA contamination. The relative quantification of mRNA expression was determined using the comparative Ct method.

### Western blotting

Cells were homogenized in RIPA buffer (Cell-Signaling Tech., US). The protein concentrations were determined using a BCA kit (Beyotime, China). Equal amounts of protein were separated on SDS-PAGE and then transferred to 0.45-μm PVDF membranes (Bio-Rad, US). The membranes were blocked in 5% bovine serum albumin (BSA) in Tris-buffered saline with Tween 20 buffer (TBST) for 2 h and then incubated overnight at 4°C with the following primary antibodies: anti-PTEN (1:1000, Cell-signaling Tech.), anti-total Akt (1:1000, Abcam), anti-phosphor ser473 Akt (1:1000, Abcam) and anti-GAPDH (1:1000, Cell-signaling Tech.), which served as a loading control. Next, the membranes were washed three times with TBST and incubated with horseradish peroxidase-conjugated secondary antibody (goat anti-rabbit IgG (1:5000) or goat anti-mouse IgG (1:5000); Cell-signaling Tech.) for 2 h at room temperature. Blots were developed using a chemiluminescence kit (Millipore, US) and exposed to X-ray film. The bands on the film were scanned and analyzed using Image J.

### Luciferase vector construction

The human *PTEN* 3′UTR containing the seed sequence of the *miR-21* binding sites was amplified by PCR and was cloned into the pMIR-REPORT vector (Ambion, USA) between the HindIII and SpeI sites to develop the pMIR-PTEN-3′-UTR luciferase vector. Seed sequences were mutated by overlap extension PCR. The mutated PTEN-3′-UTR fragment was cloned into the pMIR-REPORT vector to develop the pMIR-PTEN-mut-3′-UTR vector. A series of reporter constructs with the same 3′ terminus but different 5′ terminus of the miR-21 promoter were amplified by PCR. All of the PCR products were cloned into the plasmid pGL4-Minimal (Promega) to generate pGL4-miR-21. The mutant site was introduced into the plasmid by overlap extension PCR. The subsequent PCR product was cloned to generate pGL4-mut-miR-21. All of the constructs were confirmed by DNA sequencing.

### Luciferase activity

Cells at a density of 2×10^5^ per well were seeded into 24-well plates and cultured overnight. For the *miR-21* assay, cells were cotransfected with 0.8 μg of pMIR-PTEN-3′-UTR or pMIR-PTEN-mut-3′-UTR, 50 nM miR-21 mimic or inhibitor or scrambled oligonucleotide along with 50 ng of pRL-TK vector as an internal control using Lipofectamine 2000 reagent (Invitrogen). For the promoter assay, cells were co-transfected with 1 μg of pcNF-κB or pcDNA, 20 ng of a luciferase reporter plasmid and 10 ng of the Renilla luciferase reporter plasmid (Promega). Twenty-four hours after transfection, the cells were harvested, and the luciferase activity was determined using a dual luciferase reporter assay (Promega, USA). The results were expressed as relative luciferase activity as reported previously [15]. All experiments were repeated three times in triplicate.

### ChIP assay

The ChIP assay was performed using the Magna ChIP A/G kit (Millipore) according to the manufacturer’s recommendation. Briefly, the A549 cells were cross-linked with 1% formaldehyde (Sigma-Aldrich) for 10 min and quenched by glycine. The cross-linked cells were collected in cold PBS and sonicated for six 15-s pulses at 50-s intervals to reduce the total DNA size to 200–1000 bp. The sheared chromatin and magnetic beads were immunoprecipitated with anti-NF-κB (ab7970; Abcam, USA) or normal rabbit IgG overnight at 4°C on a rotator. Magnetic bead-antibody-chromatin complexes were washed once with a low-salt buffer, followed sequentially by high salt, LiCl and TE buffers. The chromatin complexes were eluted and incubated at 62°C for 2 h. The DNA samples were recovered using spin columns. For ChIP samples, real-time PCR was carried out using the SYBR method as described above.

### 3-(4,5-Dimethylthiazol-2-yl)-2,5-diphenyltetrazolium bromide (MTT) assay

Cells were seeded into 96-well plates at a density of 5000 cells per well. After 24 h, cells were transfected with 100 nM miR-21 mimic, miR-21 inhibitor or scrambled oligonucleotide. For combination experiments with the miR-21 mimic and pcDNA-PTEN, cells were cotransfected with pcDNA-PTEN (0.2 μg). The medium was replaced with fresh complete medium with or without cisplatin (5 μM) at 24 h post-transfection according to a previous report [16]. After an additional 24 h, 20 μl of 5 mg/mL MTT (dimethyl thiazolyl diphenyl tetrazolium; Sigma-Aldrich) was added into each well and incubated for 4 h in a humidified incubator. The supernatant was then discarded, and 200 μL of DMSO was added to each well to dissolve the formazan. The optical density (OD) was measured at 490 nm. The viability of the cells without any treatment was defined as 100%, and the viability of other groups was calculated separately from that of the sham cells.

### Statistics

Continuous variables were presented as the mean ± SD and determined using analysis of variance (ANOVA) followed by a post-hoc *t*-test using SPSS 13.0 software. The association between survival rate and *miR-21* expression was determined using multiple logistic regression analysis including the following factors: age, gender, smoker and histology. Kaplan-Meier analysis was used to evaluate the rate of event-free survival and the primary patency. Statistical significance was defined as a *P* value less than 0.05.

## Results

### *miR-21* is overexpressed in NSCLC lung tissues

**Fig 1:**
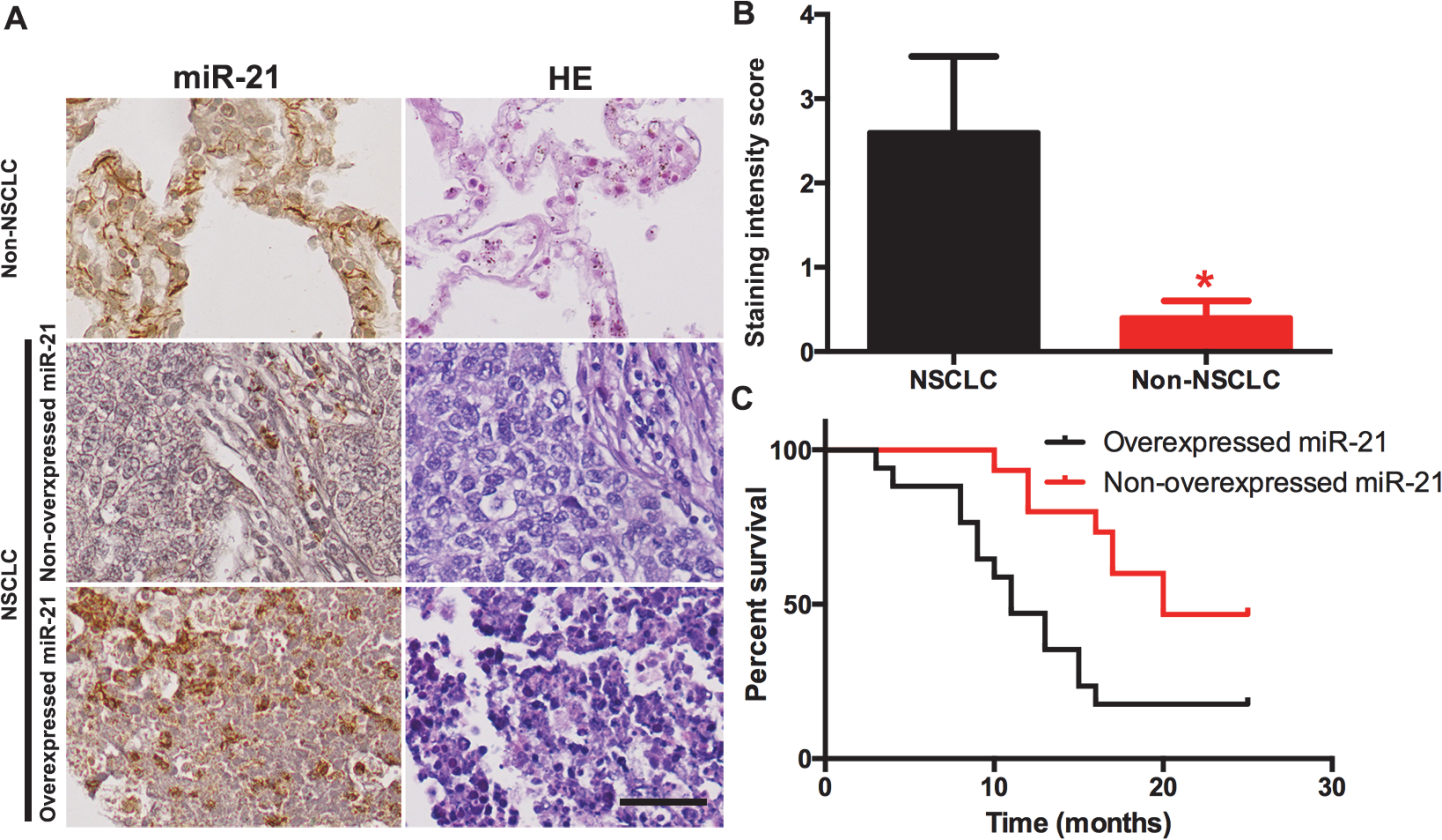
Overexpression of miR-21 in NSCLC patients. (A) ISH was used to detect the miR-21 in a TMA with 34 NSCLC and 10 normal lung samples. Left panel was ISH and right panel was HE staining. (B) The staining intensity score was higher in NSCLC patients compared with normal subjects. (C) The survival time of patients with high level of miR-21 was shorter than who with low level of miR-21. *P<0.05 as significant difference.

Lung tissues from 34 patients with NSCLC and 10 patients without NSCLC were used to make the TMA. The clinical characteristics of the subjects are shown in Table 1. The expression of *miR-21* was detected by ISH. The intensity of *miR-21* staining was significantly increased in NSCLC cells. The score was significantly increased in patients with NSCLC (Fig. 1A and B; *P* < 0.05). Moreover, the survival time was decreased in patients with NSCLC who had a high level of *miR-21* compared to those who had a low level of *miR-21* (Fig. 1C; *P*<0.05).

**Table 1:** Demographic and clinical characteristics.

	**NSCLC**			**Non-NSCLC**
	**Overexpressed miR-21**		**Non-overexpressed miR-21**	
**Age, y**		64	65	64
**Male**		8/17	9/17	5/10
**Smoker**		13/17	15/17	8/10
**Histology**				
**Adenocarcinoma**		9	9	-
**Squamous carcinoma**		3	4	-
**Bronchioloalveolar carcinoma**		5	4	-

### *miR-21* modulates the survival of A549 cells

**Fig 2:**
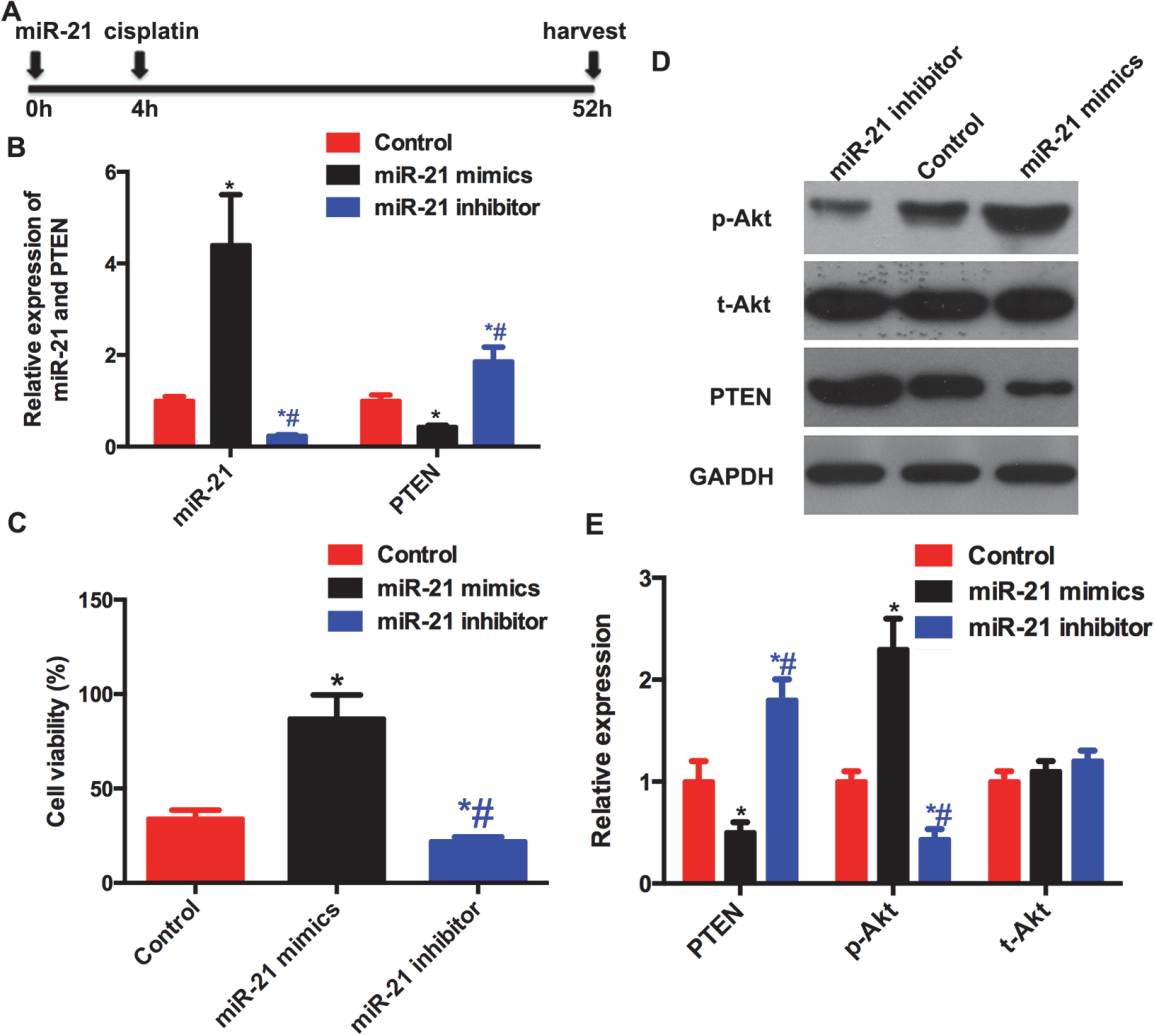
miR-21 modulated PTEN expression and cell viability in A549 cells. The cells in control, miR-21 mimics and miR-21 inhibitor were transfected with 100 nM scrambled oligonucleotide, miR-21 mimic and miR-21 inhibitor, respectively. (A) Time axis of the experiment. The scrambled oligonucleotide, miR-21 mimic or inhibitor was given 4h before cisplatin treatment (5 μM). And 48h after cisplatin treatment, the cells were harvested for further analysis. (B) The mRNA expression of PTEN was inversely related with miR-21. (C) miR-21 mimics increased the cell viability while miR-21 inhibitor decreased it when cells exposed to cisplatin treatment. (D)Western blotting of PTEN, total Akt (t-Akt) and phosphor ser473 Akt. (E) Graphic representation of PTEN, t-Akt and phosphor-Akt. miR-21 decreased the expression of PTEN and increased the ratio of phosphor-Akt. * compared with control group, # compared with miR-21 mimic group. *P*<0.05 as significant difference.

The *miR-21* mimics were used to represent exogenous *miR-21*, and the *miR-21* inhibitor was used to inhibit endogenous *miR-21*. The level of *miR-21* in the A549 cells was detected by real-time PCR (Fig. 2B). Next, we used the MTT assay to evaluate the cell viability of A549 cells when exposed to cisplatin treatment. As shown in Fig. 2C, the cell viability was significantly increased in cells treated by *miR-21* mimics compared to the control group. Inhibition of *miR-21* by its inhibitor decreased the cell viability compared to the control group.

### *miR-21* regulates *PTEN* expression by binding to its 3′-UTR

**Fig 3:**
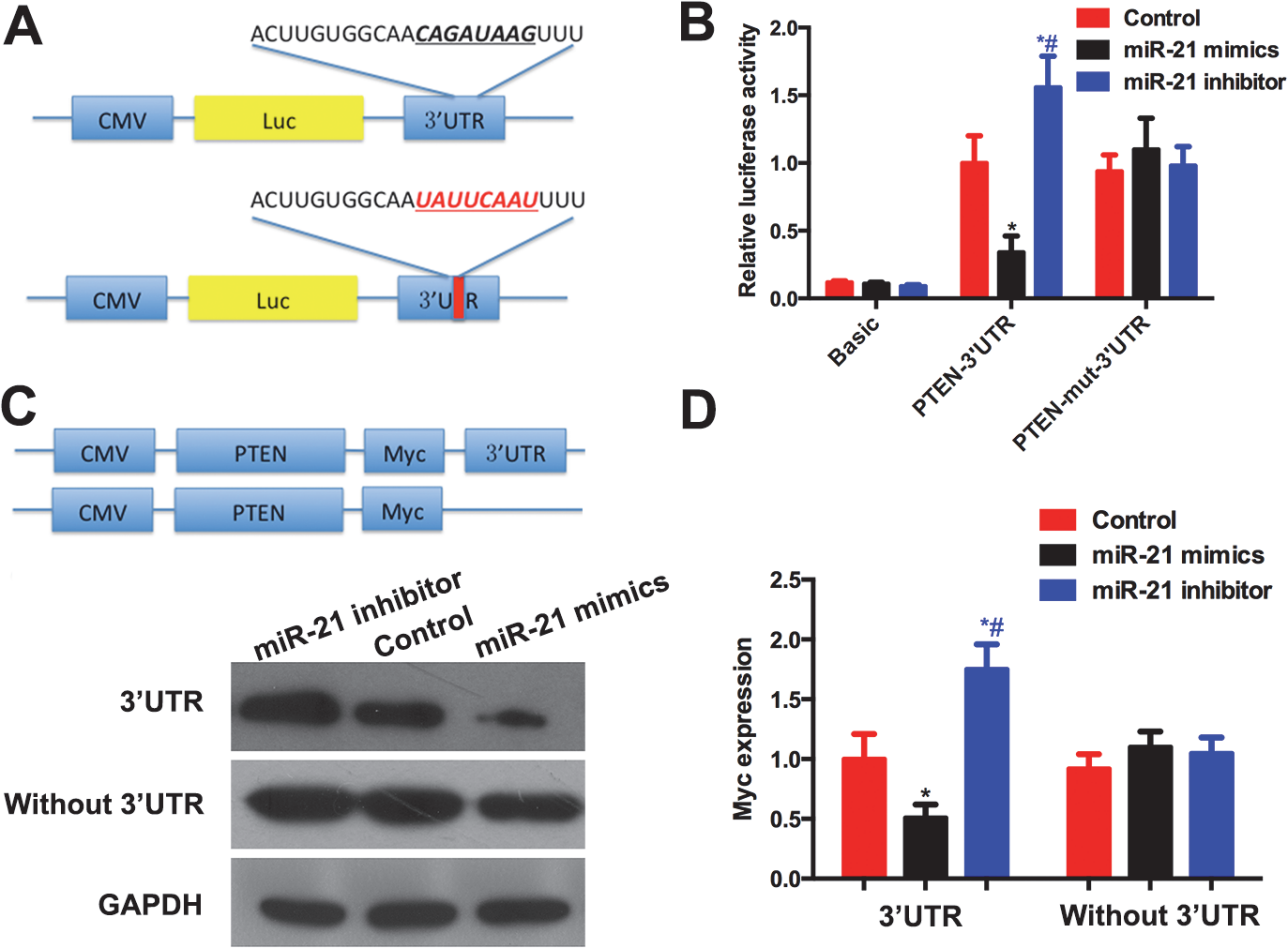
miR-21 targeting 3’UTR of PTEN. (A) Construction of 3’UTR and mutated 3’UTR of PTEN into pMIR-REPORT vector. (B) In the cells transfected with pMIR-REPORT-PTEN-3’UTR, the luciferase activity was increased by miR-21 and decreased by miR-21 inhibitor. In the cells transfected with pMIR-REPORT-PTEN-mut-3’UTR, the luciferase activity remained no significant difference among the three groups. (C) The open reading frame of PTEN with or without 3’UTR was cloned into pCS2 vector. A Myc tag was attached to PTEN to distinguish the endogenous or exogenous PTEN. (D) Graphic representation of Myc showed that overexpression of miR-21 reduced Myc expression while miR-21 inhibitor increased its expression. While the PTEN 3’UTR was knocked out, the expression of PTEN was not influenced by miR-21. * compared with control group, # compared with miR-21 mimic group. *P*<0.05 as significant difference.

A bioinformatics search demonstrated that *PTEN*, a tumor-suppressor gene, was a potential target for *miR-21* because there was a putative *miR-21*-binding seed sequence within the 3′UTR of *PTEN* mRNA (Fig. 3A). We measured the mRNA (Fig. 2B) and protein levels of PTEN (Fig. 2D) in the A549 cells with a high or low level of miR-21. An inverse correlation between *miR-21* expression and *PTEN* mRNA expression was detected. *Akt* is downstream of *PTEN*, and I also affected the activation of *Akt* (Fig. 2D and E). Compared to the control group, *miR-21* mimics decreased the luciferase activity, whereas the *miR-21* inhibitor showed a significant increase in the relative luciferase activity. When the seed sequence of the *miR-21* binding sites was mutated, the effect of *miR-21* on luciferase activity was not detected (Fig. 3B). Next, the entire open reading frame of *PTEN* with or without the *miR-21* targeting sequence in the 3′UTR was cloned into pcCS2. The *Myc* level, which represented exogenous *PTEN*, was decreased by the *miR-21* mimics in the cells transfected with pcCS2-PTEN-3’UTR, whereas there was no significant reduction in *Myc* expression by *miR-21* mimics in the cells transfected with pcCS2-PTEN (Fig. 3C and D). These results implied that *miR-21* regulated the expression of *PTEN* in A549 cells via targeting the sequence of the PTEN 3′UTR.

### NF-κB regulates the expression of miR-21 via binding its promoter

**Fig 4:**
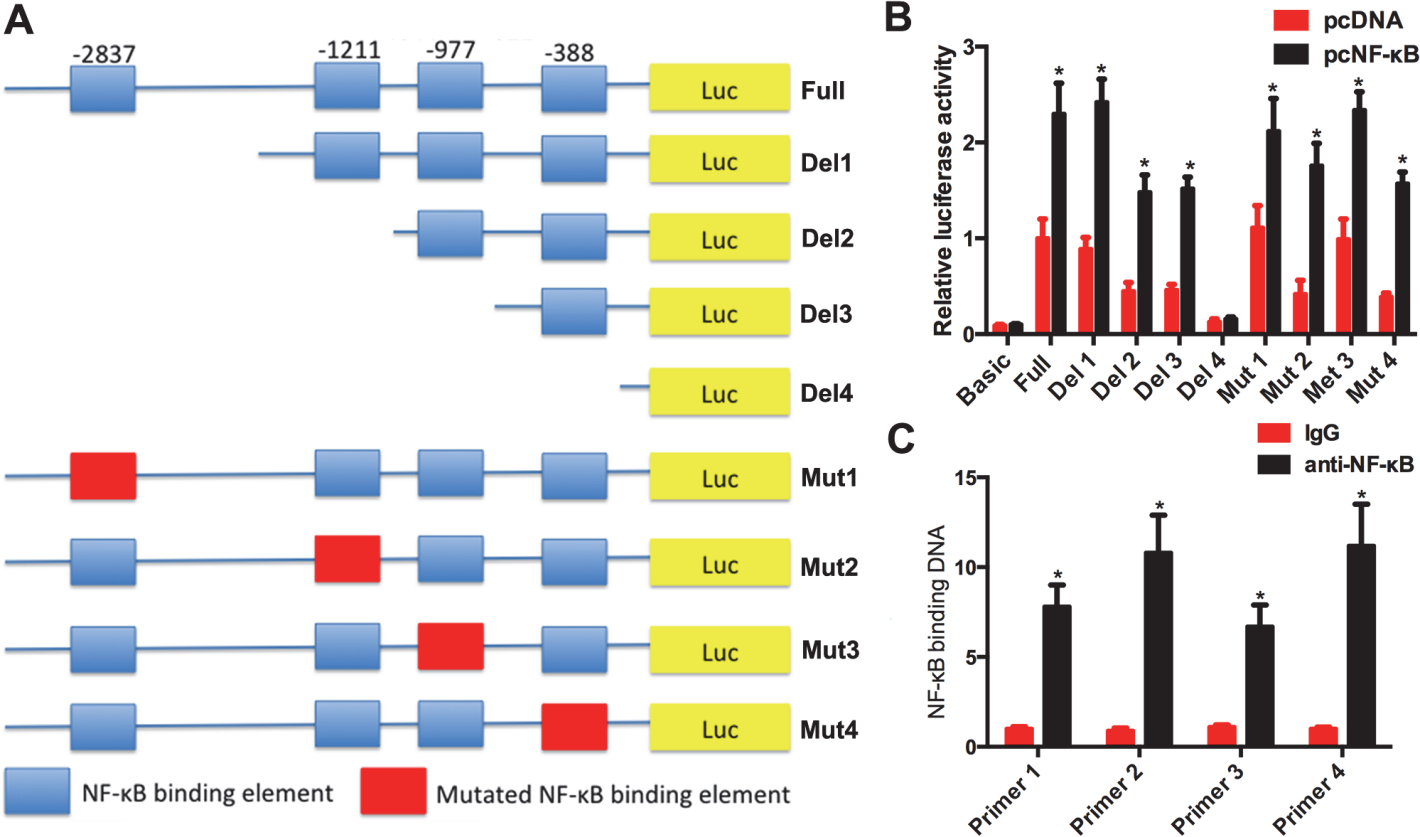
NF-κB targeting its binding elements in the miR-21 gene promoter. (A) The full length of miR-21 has four NF-κB binding elements. Different lengths of miR-21 promoter were cloned to pGL4 vector. The four elements were mutated individually and the mutated promoters were also cloned to pGL4 vector. (B) The relative luciferase activity in A549 cells cotransfected with pcNF-κB and pGL4 reporting vector. (C) ChIP realtime assay showed that NF-κB antibody bind more DNA than normal IgG. Primer 1 to 4 means the primer contained the four NF-κB binding elements from upstream to downstream. *P<0.05 as significant difference.

Given the role of *miR-21* in NSCLC, we aimed to investigate the mechanism of overexpression of *miR-21* in NSCLC. The bioinformatics analysis suggested four potential binding elements of NF-κB in the promoter region of the *miR-21* gene (Fig. 4A). The ChIP real-time assay showed that the level of NF-κB antibody binding to the *miR-21* promoter was more than that of IgG (Fig. 4C, *P*<0.05), indicating that NF-κB could directly bind to all four elements in the promoter region of the *miR-21* gene. Next, we subcloned different lengths of the *miR-21* promoter and its mutation into pGL4 and cotransfected the constructs with Renilla with or without pcNF-κB into A549 cells. As shown in Fig. 4B, pcNF-κB significantly increased the luciferase activity compared to pcDNA. When the first binding element (-2837) was deleted or mutated, there was no reduction in luciferase activity compared to the full-length promoter. When the second element (-1211) was deleted or mutated, there was significant reduction in luciferase activity. When the third element was deleted or mutated, there was no significant reduction in luciferase activity compared to when the second element was removed. However, when the binding sequences of NF-κB were all deleted or mutated, there was also significant reduction in luciferase activity compared to when the second element was deleted or mutated. These results indicated that the second and fourth binding elements might contribute to the regulation of *miR-21* transcription by NF-κB.

### The NF-κB/miR-21/PTEN pathway modulates the sensitivity of A549 cells to cisplatin

**Fig 5:**
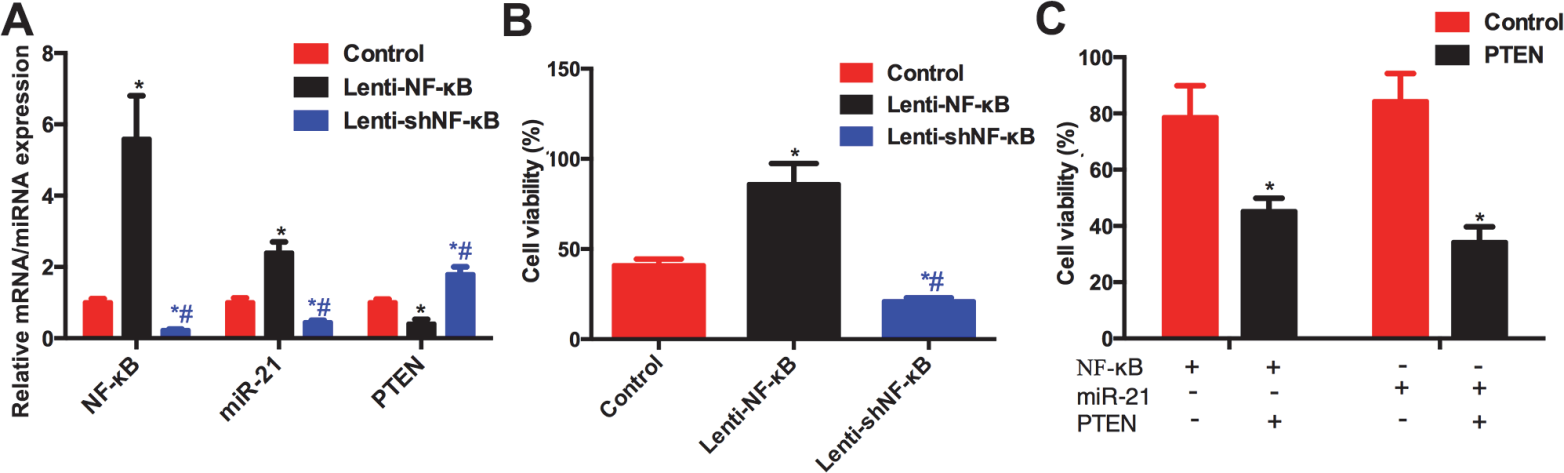
NF-κB/miR-21/PTEN pathway modulated cell viability. (A) mRNA expressions of NF-κB, miR-21 and PTEN in the cells transduced with Lenti-control, Lenti-NF-κB and Lenti-shNF-κB. The miR-21 level was significantly increased while the PTEN was decreased in the cells transduced with Lenti-NF-κB compared with control vector. The miR-21 level was significantly decreased while the PTEN was decreased in the cells transduced with Lenti-NF-κB. (B) Cell viability was increased by Lenti-NF-κB while decreased by Lenti-shNF-κB. (C) Overexpression of PTEN restored the sensitivity of A549 cells to cisplatin treatment compared with overexpression of NF-κB or miR-21. * compared with control group, # compared with Lenti-NF-κB group. *P*<0.05 as significant difference.

The level of *miR-21* was induced by overexpression of NF-κB and inhibited by Lenti-shNF-κB, which decreased the expression of NF-κB (Fig. 5A). *PTEN* expression was inversely correlated with NF-κB expression (Fig. 5A). The cell viability of A549 cells was significantly increased by Lenti-NF-κB when exposed to cisplatin treatment (Fig. 5B). In the cells transfected with pcCS2-PTEN to overexpress *PTEN*, the effects of overexpression of NF-κB or *miR-21* on the cell viability of A549 cells was inhibited (Fig. 5C).

## Discussion

In the present study, we demonstrated that *miR-21* was upregulated in patients with NSCLC, indicating a poor prognosis. Additionally, it was found that *miR-21* induced the resistance of the NSCLC cell line to cisplatin via targeting the 3′UTR of *PTEN* to decrease its expression post-transcriptionally. Moreover, NF-κB translocates into the nucleus and binds to its specific elements in the *miR-21* gene promoter to induce *miR-21* expression. Finally, modulation of the NF-κB/miR-21/PTEN pathway increased the sensitivity of NSCLC cells to cisplatin treatment. Our data might provide new insights into the pathophysiological mechanism of NSCLC and suggests a novel drug target for NSCLC.

*miR-21* has been found to be overexpressed in many types of solid tumors, including liver cancer, prostate cancer and colorectal cancer. In our present study, we extracted tissues from patients with NSCLC and control subjects to perform TMA. The ISH result detected a higher level of *miR-21* in patients with NSCLC compared to control subjects. Furthermore, the high level of *miR-21* indicated a poor prognosis for patients with NSCLC. This is supported by many previous studies. In the Markou et al. study, 48 NSCLC fresh-frozen tissue specimens were collected to detect *miR-21* expression by real-time PCR, and it was found that *miR-21* was upregulated in 16 of 29 (55.2%) relapsed patients and 15 of 23 (65.2%) deceased patients [17]. Additionally, in non-smokers, the aberrantly increased expression of *miR-21* was found to contribute to lung carcinogenesis [18]. Two meta-analyses summarized the data from different studies and concluded that overexpression of *miR-21* was an independent prognostic factor for NSCLC [19], particularly in Asian people [20].

To understand the effect of *miR-21* on NSCLC, *miR-21* mimics and its inhibitor were used, respectively, to enhance and inhibit its biological function in the A549 NSCLC cell line. It was found that exogenous *miR-21* promoted cell survival when exposed to cisplatin treatment, whereas *miR-21* inhibition led to the opposite function. These findings were validated by one study, which showed that elevated *miR-21* increased the resistance of A549 cells to platinum, whereas reduced *miR-21* decreased this resistance [21]. Based on the above findings, *miR-21* might be a potential therapeutic target for NSCLC. The underlying mechanism of cisplatin resistance of NSCLC is complicated, and *miR-21* also has multiple targets according to the bioinformatics analysis. To explore the effect of I on NSCLC before it advances further into the clinical application stage, two essential questions persist: how I affects the outcome of NSCLC, and how I is upregulated in NSCLC.

Bioinformatics studies suggest a target site of *miR-21* in the 3′UTR of *PTEN* mRNA. I is a ubiquitous tumor-suppressor gene, and its deregulation results in many solid tumors. In NSCLC, I has been implicated as a key contributor to pathogenesis and tumor growth [22]. *PTEN* loss has been recognized as the mechanism of gefitinib resistance in NSCLC [23]. Targeted deletion of *PTEN* leads to the development of NSCLC [24]. Hence, it was assumed that *miR-21*, which inhibits *PTEN* by targeting its 3′UTR, would affect the NSCLC pathological process. We initially identified an inverse relationship between *miR-21* and *PTEN* in A549 cells. We then cloned the 3′UTR of *PTEN* into the pMIR-REPORT luciferase reporter and found that *miR-21* mimics significantly decreased luciferase activity when cotransfected with pMIR-REPORT-PTEN-3’UTR, whereas no change in luciferase activity was noted when *miR-21* mimics were cotransfected with pMIR-REPORT-PTEN without the 3′UTR. Previous studies have also supported this finding [25,26].

On one hand, *miR-21* regulated the expression of *PTEN*; on the other hand, it was regulated by other factors. Some miRNAs have their own promoters, whereas others share a promoter with other miRNAs for the miRNA gene located in the intron of the host gene. Whether miRNAs have their own promoters or share promoters, their expression can be regulated by certain transcription factors, including p53 and NF-κB, which target their promoters. There are four binding elements of NF-κB in the *miR-21* gene promoter [27]. Hence, it is possible that *miR-21* could be regulated by NF-κB targeting its binding elements. Our ChIP assay confirmed that NF-κB could directly bind to all four elements in the promoter of the *miR-21* gene. However, only the elements located at -1211 and -404 of the *miR-21* gene promoter had biological function according to the luciferase activity assay. Our data were partially supported by those of other studies. In prostate cancer cells, the interferon-induced *miR-21* expression required NF-κB/p65 recruitment to the *miR-21* promoter [27]. In the human breast MDA-MB-231 and colorectal HCT116 tumor cell lines, NF-κB was also found to bind to the *miR-21* gene promoter [28]. In NSCLC, NF-κB was first identified to regulate the expression of *miR-21* by binding to its elements in the *miR-21* gene promoter. We also found that NF-κB-induced *miR-21* expression modulates its target gene, *PTEN*, the reduced expression of which promotes cell survival. Overexpression of *PTEN* in A549 cells could reverse the effect of *miR-21* and NF-κB to restore the sensitivity of A549 cells to cisplatin.

In conclusion, our observations indicated that the NF-κB/miR-21/PTEN pathway contributes to the resistance of NSCLC to cisplatin treatment.
